# AK2 is an AMP-sensing negative regulator of BRAF in tumorigenesis

**DOI:** 10.1038/s41419-022-04921-7

**Published:** 2022-05-18

**Authors:** Hyunjoo Kim, Muhah Jeong, Do-Hyeong Na, Shin-Hyeon Ryu, Eun Il Jeong, Kwangmin Jung, Jaemin Kang, Ho-June Lee, Taebo Sim, Dae-Yeul Yu, Hee Chul Yu, Baik-Hwan Cho, Yong-Keun Jung

**Affiliations:** 1 School of Biological Science, Seoul National University, Gwanak-gu, Seoul, 08826 Korea; 2 Departments of Discovery Oncology, Genentech, Inc., South San Francisco, CA 94080 USA; 3 Chemical Kinomics Research Center, Korea Institute of Science and Technology, Seoul, 02792 Korea; 4 Aging Intervention Research Center, Korea Research Institute of Bioscience and Biotechnology, Daejeon, Korea; 5 Department of Surgery, Chonbuk National University Medical School, Jeonju, 561-180 Korea

**Keywords:** Tumour-suppressor proteins, Diagnostic markers, Extracellular signalling molecules

## Abstract

The *RAS–BRAF* signaling is a major pathway of cell proliferation and their mutations are frequently found in human cancers. Adenylate kinase 2 (AK2), which modulates balance of adenine nucleotide pool, has been implicated in cell death and cell proliferation independently of its enzyme activity. Recently, the role of AK2 in tumorigenesis was in part elucidated in some cancer types including lung adenocarcinoma and breast cancer, but the underlying mechanism is not clear. Here, we show that AK2 is a BRAF-suppressor. In in vitro assays and cell model, AK2 interacted with BRAF and inhibited BRAF activity and downstream ERK phosphorylation. Energy-deprived conditions in cell model and the addition of AMP to cell lysates strengthened the AK2-BRAF interaction, suggesting that AK2 is involved in the regulation of BRAF activity in response to cell metabolic state. AMP facilitated the AK2–BRAF complex formation through binding to AK2. In a panel of HCC cell lines, AK2 expression was inversely correlated with ERK/MAPK activation, and AK2-knockdown or -knockout increased BRAF activity and promoted cell proliferation. Tumors from HCC patients showed low-AK2 protein expression and increased ERK activation compared to non-tumor tissues and the downregulation of AK2 was also verified by two microarray datasets (TCGA-LIHC and GSE14520). Moreover, AK2/BRAF interaction was abrogated by RAS activation in in vitro assay and cell model and in a mouse model of HRAS^G12V^-driven HCC, and AK2 ablation promoted tumor growth and BRAF activity. AK2 also bound to BRAF inhibitor-insensitive BRAF mutants and attenuated their activities. These findings indicate that AK2 monitoring cellular AMP levels is indeed a negative regulator of BRAF, linking the metabolic status to tumor growth.

## Introduction

The RAS–RAF–MEK–ERK protein kinase cascade is a major signaling pathway that stimulates cell proliferation in response to extracellular mitogenic signals [1, 2]. Not surprisingly, this pathway is frequently activated in human cancers [3, 4]. The complexity of this pathway is compounded by the presence of multiple components [3]: three RAS proteins (H-, N-, and K-RAS), three RAF proteins (A-, B-, and C-RAF), two MEK versions (MEK1 and -2), and two ERK proteins (ERK1 and 2) [5, 6]. Recent studies showed that up to 15 and 8% of human cancers harbor an activating mutation in the proto-oncogene *RAS* and *BRAF* [7]. Therefore, small-molecule inhibitors specific to RAS and BRAF have been actively searched. However, the development of RAS inhibitors for cancer therapeutics has been unsuccessful. In contrast, BRAF inhibitors, e.g., PLX4032 [8], have been approved for the treatment of some cancers [9]. Although BRAF-selective inhibitors block ERK signaling in tumors with *BRAF* mutation, they paradoxically activate ERK signaling and accelerate the growth of tumors with wild-type (WT) BRAF or with *RAS* mutations [10–12], thereby limiting the usefulness of BRAF inhibitors as a cancer therapy. Hence, it is worth identifying clinically effective WT and mutant-BRAF–targeting regulators in the RAS-BRAF signal, including but not limited to 14-3-3, AMPK [13], or BRAF-interacting proteins.

Adenylate kinase 2 (AK2) is known to govern the balance of the synthesis and consumption of the adenine nucleotide pool [14, 15]. In addition to original role of AK2, recent studies revealed that AK2 is a multi-functional protein; human AK2 deficiency is responsible for reticular dysgenesis, the most severe form of inborn human severe combined immunodeficiencies (SCID) [16, 17]. AK2 forms a novel apoptotic complex containing FADD and caspase 10 in the cytosol during apoptosis [18], and stimulates dephosphorylation of phospho-FADD_Ser194_ via DUSP26 in growing cells [19]. Besides, it was also shown that AK2 is methylated with the development of antituberculosis drug-induced liver injury (ATLI) [20] and overexpressed in lung adenocarcinoma [21] and in metastatic pancreatic endocrine neoplasms, which preferentially metastasized to liver [22]. Despite accumulating evidence supporting the role of AK2 in tumorigenesis, a correlation between AK2 and common driver genes in cancers, including MAPK pathway genes, remains poorly understood.

Hepatocellular carcinoma (HCC) is the commonest primary liver tumor and has a poor prognosis [23, 24]. In HCC, RAS and RAF gene mutations are rare compared to melanoma, pancreas- and colon-cancers; less than 10% of HCC patients harbor RAS mutations, while Colombino et al. reported V600E mutation on BRAF in 23% of HCC patients from South Italy [25]. On the other hand, RAS/MAPK pathway is activated in 50–100% of HCC patients and shows a correlation to poor prognosis [26], and RAS-BRAF pathway was reported to affect HCC [25, 27, 28]. In accordance to this, Sorafenib, a multi-kinase inhibitor, which blocks RTKs and RAF isoforms leading to inactivation of MAPK pathway, and Lenvatinib, an oral inhibitor of RTKs, were the first-line approved drugs for the treatment of advanced HCC [29, 30]. RAS overexpression and activation also occur in HCCs; this elevation is associated with poor prognosis in patients [23, 31–35]. In contrast to the inhibitors regulating MAPK pathway, the drugs targeting most prevalent oncogenes and tumor suppressors observed in HCCs, including TERT, CTNNB1, TP53, and AXIN1, are not clinically actionable [36]. These observations potentiated the importance of the RAS-RAF-MAPK pathway in HCC development and led us to identify a novel regulator of MAPK pathway in liver tumorigenesis.

Here, we identified AK2 as an AMP-sensing negative regulator of BRAF and delineated the tumor suppressive role of AK2 in liver tumorigenesis of *HRAS*^G12V^-transgenic (Tg) mice.

## Materials and methods

### Identification of BRAF-binding proteins by LC-MS/MS

HEK293T cells were transfected with PCD3.1, 3xFlag-BRAF or 3x-Flag-AK2 for 24 h and lysed with immunoprecipitation (IP) lysis buffer (50 mM Tris, pH 6.8, 30 mM NaCl, 1% Triton X-100 and 1 mM PMSF) with sonication. Cell lysates were then incubated with anti-Flag antibody-conjugated agarose beads (Sigma-Aldrich Cat# A2220) for 12 h and loaded into SDS-PAGE. Bands of interests in BRAF- or AK2-overexpressed lane were cut-out, destained (30% acetonitrile, 1 h), reduced (20 mM DTT, 10 m), alkylated (55 mM iodoacetamide, 1 h), and trypsinized (0.5 μg, overnight, 37 °C). The peptides were analyzed using Orbitrap Fusion Lumos Tribrid MS (Thermo Fisher Scientific) coupled with nanoAcquity system (Waters). Full resolutions of mass spectrometry were set to 60k at m/z 200 with 350–1500 mass range.

### In vitro BRAF kinase assay

Catalytic domain of GST-BRAF protein was purchased (Merck Cat#14-530). Kinase reactions were performed at room temperature in Base Reaction buffer [20 mM HEPES (pH 7.5), 10 mM MgCl2, 1 mM EGTA, 0.02% Brij35, 0.02 mg/ml BSA, 0.1 mM Na3VO, 2 mM DTT, 1% DMSO] with 10 nM BRAF (Merck Cat# 14-530), 1 μM MEK1 (Merck Cat#14-429), and/or purified proteins at a final volume of 20 μl. AMP, ADP, or ATP were added at the concentrations given in the figure legends. After 30 min, the kinase reaction was initiated by adding 10 μCi [γ^32^P] ATP (PerkinElmer) and progressed by incubating the reaction mixture at room temperature for 2 h.

### Animal studies

All mice were approved by and handled in accordance with the guidelines of the Institutional Animal Care and Use Committee at UTSW. The protocols were certified by the Institutional Animal Care and Use Committee (IACUC) of Seoul National University. *HRAS*^G12V^ Tg mice has been previously described [37, 38] to induce liver cancer. *HRAS*-Tg;*AK2*-KO mice were generated by mating *AK2*^+/−^ knockout mice [19] to *HRAS* Tg mice. No statistical methods were used to predetermine sample size, but the sample size analyzed in this study is similar to the previous publications (e.g., ref. [39]).

Detailed descriptions of materials and methods are included in ‘Supplementary Information.’

## Results

### AK2 interacts with BRAF to inhibit the BRAF kinase activity

**Fig. 1:**
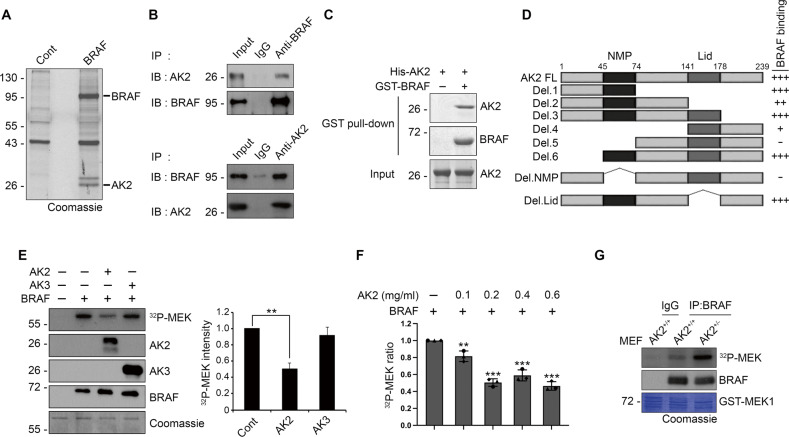
AK2 interacts with BRAF, restraining the BRAF kinase activity. **A** HEK293T cells were transfected with empty (Cont) or 3xFlag-BRAF (BRAF) for 24 h and cell extracts were incubated with anti-Flag antibody-conjugated to agarose bead in immunoprecipitation (IP) lysis buffer for 12 h. The immunoprecipitated proteins were then resolved by SDS-PAGE (Coomasie blue) and the bands of interests were sliced into pieces for LC-MS/MS. **B** Cellular interaction between BRAF and AK2. Hep3B cell extracts were subjected to immunoprecipitation (IP) assay with anti-BRAF (upper) or anti-AK2 (lower) antibody. **C** AK2 binds to BRAF in vitro. Purified GST-BRAF and His-AK2 proteins (each 1 μg ml^−1^) were combined together for 1 h and then subjected to GST pull-down assays. The pulled samples and input were analyzed by western blotting. **D** The region encompassing a.a. 45–74 of AK2 is essential for AK2-BRAF association. A schematic diagram of AK2 deletion mutants and a summary of their binding to BRAF are described. The blots in Fig S1E, F were quantified their signals and the values are represented; +++ (> 0.8), ++ (0.5–0.8), + (0.5–0.2), − (<0.2). **E** AK2 inhibits BRAF activity in vitro. GST-BRAF protein (10 nM) was incubated with either purified His-AK2 (1 μM) or His-AK3 (1 μM) protein. The kinase reaction using GST-MEK1 (1 μM) as a substrate was progressed for 2 h in the presence of 10 μM Ci [γ-^32^p] ATP. MEK phosphorylation was visualized by autoradiography (left upper) and the signals of [γ-^32^p] were quantified by densitometric analysis (right). Proteins utilized in the assay were confirmed by western blotting and Coomassie staining (left). Values are presented as mean ± SD (*n* = 3; **p < 0.01). **F** Dose-dependent inhibition of BRAF activity by AK2. BRAF protein (10 nM) was incubated with the indicated concentrations of AK2 proteins. The kinase reaction was performed as described in **E**. Graph bars, error bars, and dots respectively represent mean ± SD and the individual values of 3 independent experiments. Significance was determined by one-way ANOVA with dunnett’s multiple comparisons test (*p < 0.05, **p < 0.01, ***p < 0.001). **G** BRAF proteins were isolated by immunoprecipitation (IP) assay using anti-BRAF antibody from wild-type (AK2^+/+^) or *AK2* knockout (AK2^+/−^) MEF cells and assayed for the kinase activity with γ-^32^p-MEK1.

To identify proteins that can regulate the RAF–MEK–ERK signaling, we analyzed the composition of BRAF protein complexes in the human embryonic kidney cells, HEK293T. Using a mass spectrometry approach following FLAG-bead purification, we identified AK2 as a candidate for a BRAF–associated protein (Fig. 1A, Fig. S1A, and Supplementary Table 1). Raf-1, heat shock protein 60, and BRAF substrates, previously identified as BRAF-associated proteins, were detected in our purification assays (Supplementary Table 1). We confirmed the interaction between endogenous-BRAF and AK2 in BRAF WT-carrying tumor cell lines including NCTC-1469 normal hepatocyte cells (Fig. 1B and Fig. S1B). In addition, in vitro binding assays revealed that recombinant BRAF protein directly interacted with a purified AK2 protein (Fig. 1C). Although AK2 localized in the both cytosol and mitochondria and BRAF was mainly located in the cytosol, only cytosolic AK2 interacted with BRAF (Fig. S1C, D).

By using various AK2 deletion mutants, we determined a region of AK2 protein responsible for the interaction with BRAF. An immunoprecipitation assay revealed that AK2 N-terminus-deletion mutants Del.4 and Del.5, but not C terminus-deletion mutants Del.1, Del.2, Del.3 and lid mutants, impaired BRAF/AK2 interaction (Fig. 1D and Fig. S1E, F). Further, the AK2 Del.NMP mutant lacking the NMP domain (amino acid residues 45–74), which contains an AMP-binding motif, did not bind to BRAF (Fig. 1D and Fig. S1F, G). These data indicate that the NMP domain of AK2 is required for BRAF-binding. In agreement, the N-terminus-deletion mutants Del.6 including AK2 NMP domain efficiently interacts with BRAF.

We next tested whether AK2 binding affects the enzymatic activity of BRAF. In an in vitro enzymatic assay that utilized MEK as a BRAF substrate, we measured the BRAF kinase activity after incubation with a recombinant His-AK2 protein. Of note, incubation with purified AK2 protein, but not with AK3 mitochondrial protein [14], impaired the ability of BRAF to phosphorylate MEK (Fig. 1E). This inhibition of BRAF activity by AK2 was dose-dependent (Fig. 1F and Fig. S1H). In addition, we addressed whether this regulation of BRAF activity could be recapitulated in cells. Immunopurified BRAF complexes from mouse embryonic fibroblasts (MEFs) revealed that the BRAF kinase activity was higher in heterozygous *AK2* knockout (*AK2*^+/−^) MEFs than in WT MEFs (Fig. 1G). Otherwise, AK2 did not bind to CRAF (Fig. S1I) and the kinase activity of CRAF was not affected when co-incubated with purified AK2 or AK3 protein (Fig. S1J). Collectively, AK2 suppresses the enzymatic activity of BRAF, not CRAF.

### AMP enhances the binding of AK2 to BRAF

**Fig. 2:**
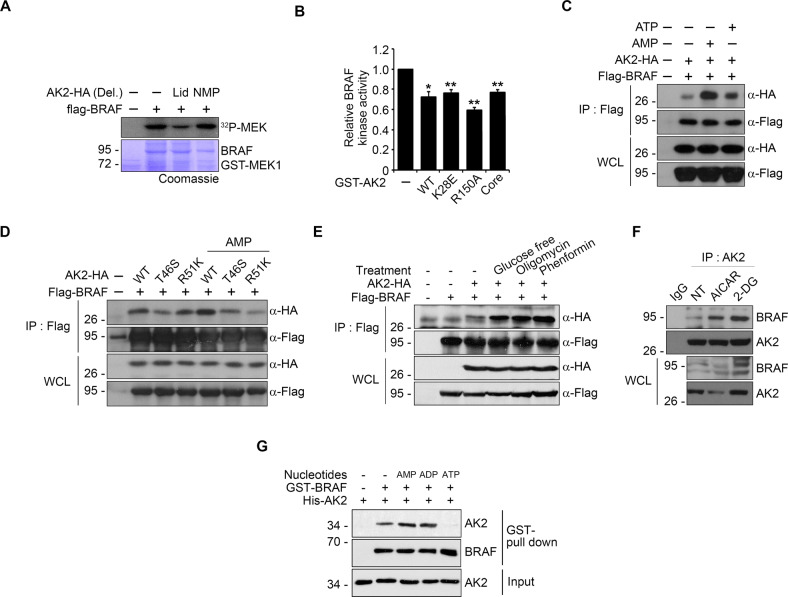
AMP potentiates the ability of AK2 to inhibit BRAF activity via enhanced affinity between AK2 and BRAF. **A** AK2 NMP domain is crucial to modulate BRAF kinase activity. HEK293T cells were transfected with Flag-BRAF and HA-AK2 deletion mutants for 24 h and subjected to kinase assay after immunoprecipitation (IP) assay using anti-FLAG agarose-beads. **B** AK2 activity-dead mutants suppresses BRAF activity in vitro. BRAF protein (10 nM) was incubated with GST-AK2 WT (1 μM) or mutant proteins (GST-AK2 K28E, R150A, and Core, 1 μM). Values are presented as mean ± SD (*n* = 3; *p < 0.05, **p < 0.01, ***p < 0.001). **C** AMP enhances the AK2–BRAF binding. After transfection of HEK293T cells with Flag-BRAF and AK2-HA, cell extracts were incubated for 12 h with 100 μM AMP or ATP and subjected to immunoprecipitation (IP) assay. **D** AMP binding-defective AK2 mutants lose AMP-stimulation of AK2-binding to BRAF. After transfection of HEK293T cells with BRAF and either AK2 WT or mutants (T46S or R51K), cell lysates were incubated with 100 μM AMP and subjected to immunoprecipitation assay. **E** Energy deprivation conditions enhance AK2-HA–Flag-BRAF binding. After transfection of HEK293T cells with Flag-BRAF and AK2-HA, cells were incubated under the following condition; glucose-free DMEM (4 h), oligomycin (1 μg/ml, 2 h), or phenformin (40 μM, 2 h). Cell lysates were subjected to immunoprecipitation (IP) assay. **F** Energy deprivation promotes the interaction between endogenous AK2 and BRAF. SK-Hep1 cells were treated with 2-DG (25 mM) or AICAR (1 mM) for 12 h and then subjected to immunoprecipitation (IP) assay. **G** AMP or ADP potentiates the AK2–BRAF binding in vitro. Purified GST-BRAF and His-AK2 proteins (each 1 μg ml^−1^) were combined together for 1 h in the presence or absence of AMP, ADP, or ATP and then subjected to GST pull-down assay. The pulled-samples and inputs were analyzed by western blotting.

As the NMP domain of AK2 contains AMP-binding motif and is important for AK2-BRAF interaction, we tested whether the NMP domain was essential for AK2-mediated inhibition of BRAF kinase activity. In BRAF kinase assays, AK2 Del.NMP mutant did not show any inhibitory effects on the kinase activity of BRAF (Fig. 2A). However, BRAF kinase activity was also inhibited by catalytically inactive AK2 mutants (K28E or R150A) or by an AK2 core deletion mutant as much as WT AK2 (Fig. 2B). Thus, enzymatic activity of AK2 is not likely to affect BRAF activity.

In the photoaffinity labeling assay, we found that 8-azido-AMP or 8-azido-ATP was equally incorporated into both BRAF and AK2 proteins in vitro (Fig. S2A, B). On the contrary, these labeling patterns were changed when BRAF and AK2 proteins were incubated together. AK2 bound better to 8-azido-AMP than to 8-azido-ATP, whereas BRAF bound better to 8-azido-ATP (Fig. S2C). Immunoprecipitation assays revealed that addition of AMP to cell lysates robustly increased the amount of AK2^WT^ bound to BRAF but not AK2^T46S^ and AK2^R51K^, containing a mutation on NMP domain [40] (Fig. 2C, D). Furthermore, AMP/ATP ratio-increasing conditions including glucose-free medium, oligomycin, phenformin, or 2-DG strengthened the AK2-BRAF interaction (Fig. 2E, F and Fig. S2D). Likewise, in vitro binding assays showed that the AK2–BRAF interaction was strengthened by AMP and ADP but weakened by ATP (Fig. 2G). Thus, increased AMP level, which represents energy deprivation in cells, strengthens the AK2–BRAF interaction via its binding to AK2.

### AK2 deficiency amplifies the BRAF signal and is detected in cancer cells and tissues

**Fig. 3:**
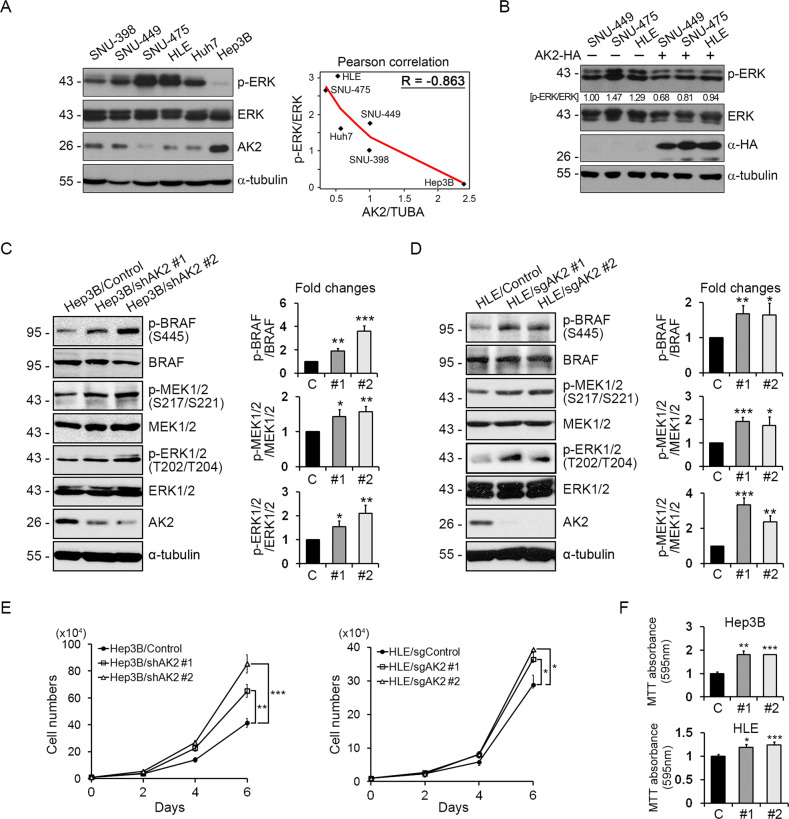
AK2 loss enhances BRAF signaling to increase cell-proliferation and -migration in liver cancer cell lines. **A** AK2 expression is down-regulated in liver tumor cell lines showing high p-ERK level. A panel of liver tumor cell lines (BRAF WT) was analyzed with western blotting (left). Total **ɑ**-tubulin was used as a loading control. The graph shows Pearson correlation between AK2/TUBA and p-ERK/ERK ratios. **B** Reconstitution with AK2 decreases p-ERK level. SNU-449, SNU-475, and HLE tumor cell lines were transfected with AK2-HA and assessed with western blotting. Total **ɑ**-tubulin was used as a loading control. The p-ERK/ERK ratios are denoted on the blots. **C**, **D** AK2 depletion enhances BRAF signaling in HCC cells. Hep3B/Control, Hep3B/shAK2, HLE/Control, and HLE/sgAK2 cells were analyzed with western blotting (left). Total **ɑ**-tubulin was used as a loading control. The signals on the blots were quantified and bars represent mean ± SD (*n* = 3) (right). **E** Hep3B and HLE cells and AK2 knockdown cells were monitored for cell proliferation for 6 days (10^4^ cells at day 0). Values represent mean ± SD (*n* = 3). **F** Proliferations of Hep3B and HLE cells and AK2 knockdown cells were analyzed with MTT assay. Values obtained by 595 nm absorbance were relatively compared to control. Bars represent mean ± SD (*n* = 3).

In mammals, AK2 is ubiquitously expressed, with the most prominent expression in the liver. We, therefore, analyzed AK2 expression in a panel of 14 human HCC cell lines, all of which express WT BRAF. We found that the AK2 was relatively under-expressed in 10 HCC cell lines (SNU-398, SNU-449, SNU-475, HLE, Huh7, Chang liver, SK-Hep1, HepG2, SNU-354, and SNU-423), and highly expressed in 4 HCC cell lines (Hep3B, SNU-368, SNU-182, and SNU-387) compared to NCTC1469 normal hepatocyte cells (Fig. 3A and Fig. S3A). Subsequently, we found a marked inverse correlation between p-ERK and AK2 level in HCC cell lines (Fig. 3A). Conversely, restoration of AK2 expression in SNU-449, SNU-475, and HLE cells reduced the level of p-ERK (Fig. 3B) and cell proliferation (Fig. S3B).

We next assessed our hypothesis that AK2 negatively regulates the BRAF activity in growing tumor cells. We found that the phosphorylation of BRAF at Ser445, which is essential for BRAF activation [41], MEK1/2, and ERK1/2 was higher in AK2 KD Hep3B cells and AK2 KO HLE cells than their control cells (Fig. 3C, D). Accordingly, ectopic expression of AK2, not AK3, in NIH3T3 cells carrying an introduced HRAS^G12V^ oncogene suppressed ERK activation (Fig. S3C) and attenuated a BRAF-induced reporter activity of Elk (Fig. S3D). In addition, cell proliferation rates increased by AK2 depletion and this increase was rescued by AK2 reconstitution in both Hep3B cells and HLE cells (Fig. 3E, F and Fig. S3E, F). BRAF-inhibitor PLX4032 also caused acceleration of ERK activation and cell proliferation in AK2-deficient Hep3B cells (Fig. S3G, H). These results indicate that AK2 restrains the BRAF activity for the control of cell proliferation in HCC cells.

**Fig. 4:**
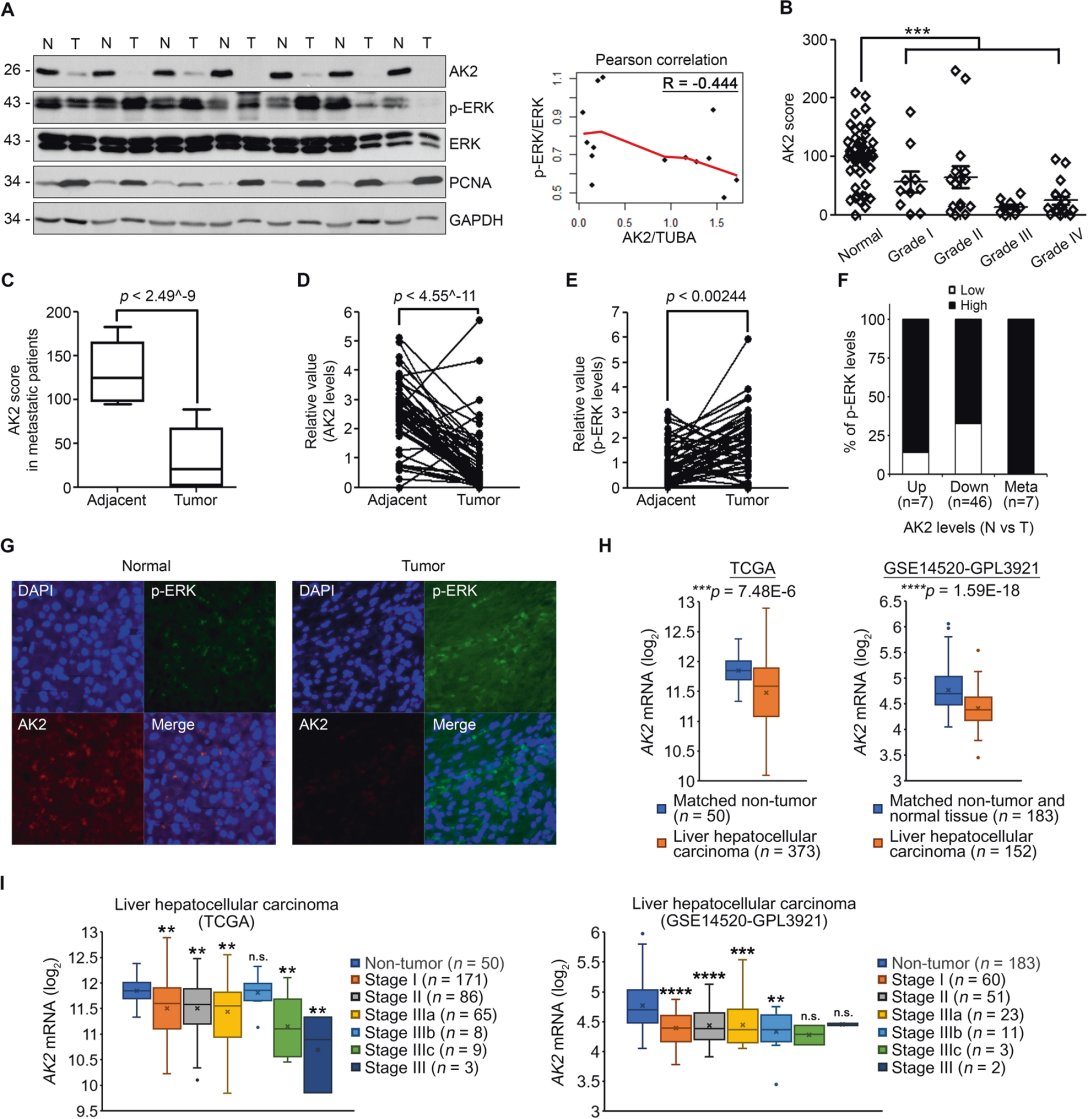
AK2 loss and ERK1/2 phosphorylation show an inverse correlation in human HCC patients. **A** Loss of AK2 correlates with increased p-ERK level in series of human liver cancers. Tissue extracts prepared from liver tumors (T) and surrounding hepatic tissues (N) were examined with western blotting (left). Total GAPDH was used as a loading control. The graph shows Pearson correlation between fold changes of AK2/TUBA and p-ERK/ERK levels (right). **B** Quantification of relative AK2 levels in (A). Each symbol represents an individual patient. (*n* = 53; ***p < 0.001). **C** Differences in AK2 level, as in (**B**), according to metastatic state of human liver tumors (by Mann–Whitney U test). Data are presented as Turkey box-and-whisker plots. Values are presented as mean ± SD (*n* = 53; ****p < 0.0001). **D**, **E** Statistical analysis showing relative levels of AK2 and p-ERK in 53 paired tumor and adjacent samples. Statistical analysis was performed by paired Student’s t-test. Values are presented as mean ± SD (*n* = 53; *p < 0.05, **p < 0.01, ***p < 0.001, ****p < 0.001). **F** Summaries of AK2 expression profile and inverse correlations between AK2 expression and p-ERK level in liver specimens. Data were first organized by AK2 expression levels (Up, Down) in tumors compared to adjacent tissues and then further separated into two categories according to p-ERK levels (Low, High) in tumors. In addition, metastasized HCC tissues, which had shown significant reduction in AK2 levels, were also analyzed as separated category. **G** Immunohistochemistry with anti-AK2 and anti-p-ERK antibodies was performed on the paraffinized-tissue slides and observed under confocal microscope. **H** Comparison of AK2 expressions in HCC patient samples. HCC datasets (TCGA-LIHC and GSE14520-GPL3921) containing microarray data were analyzed for their AK2 mRNA expressions between tumor and the matched non-tumor tissues. Box-and-whisker plots were statistically analyzed by paired Student’s t-test (*p < 0.05, **p < 0.01, ***p < 0.001, ****p < 0.0001). **I** HCC datasets from (**H**) were sorted by tumor stages. *P* values were obtained by one-way analysis of variance (ANOVA) followed by Dunnett’s test for multiple comparison (*p < 0.05, **p < 0.01, ***p < 0.001, ****p < 0.0001).

We extended our analysis to human HCC tissues. We examined AK2 expression in 53 human HCC tissue samples and found that the AK2 level remarkably decreased in 46 human HCC samples (86.8% of all samples), as compared to their surrounding nontumorous liver tissue (Fig. 4A–D and Fig. S4A). In particular, a marked reduction in AK2 levels was observed in the metastasized HCC tissues (Fig. 4C). Consistent with the results observed in the HCC cell lines, p-ERK levels were elevated in the AK2-underexpressing cancer tissues (Fig. 4E and Fig. S4A). Among all 46 AK2-underexpressing cancer tissue samples, p-ERK levels were high in 32 samples (69.6%) and low in 14 tissue samples (30.4%) (Fig. 4F). Immunohistochemical assays confirmed that tumor samples with a strong p-ERK signal manifested weak AK2 staining (Fig. 4G). Thus, there was an inverse correlation between AK2 and p-ERK levels in human liver cancer tissue samples.

Furthermore, we analyzed two HCC datasets; The Cancer Genome Atlas HCC dataset (TCGA-LIHC) which contains microarray data from 373 patients; GSE14520-GPL3921 HCC dataset which also contains gene expression profiles from 152 patients and normal tissues. As expected, AK2 expression was significantly downregulated in HCC tumor tissues compared to matched non-tumor or normal tissues in the both datasets (Fig. 4H). In specific, low expression of AK2 was validated from early- to late-stages (TNM) of HCC patients in the both datasets, with few exceptions (Fig. 4I). Together, downregulated-AK2 expression is related to tumorigenesis in HCC patients.

### *AK2* haploid deficiency promotes *HRAS*^G12V^-driven HCC formation via BRAF in mice

**Fig. 5:**
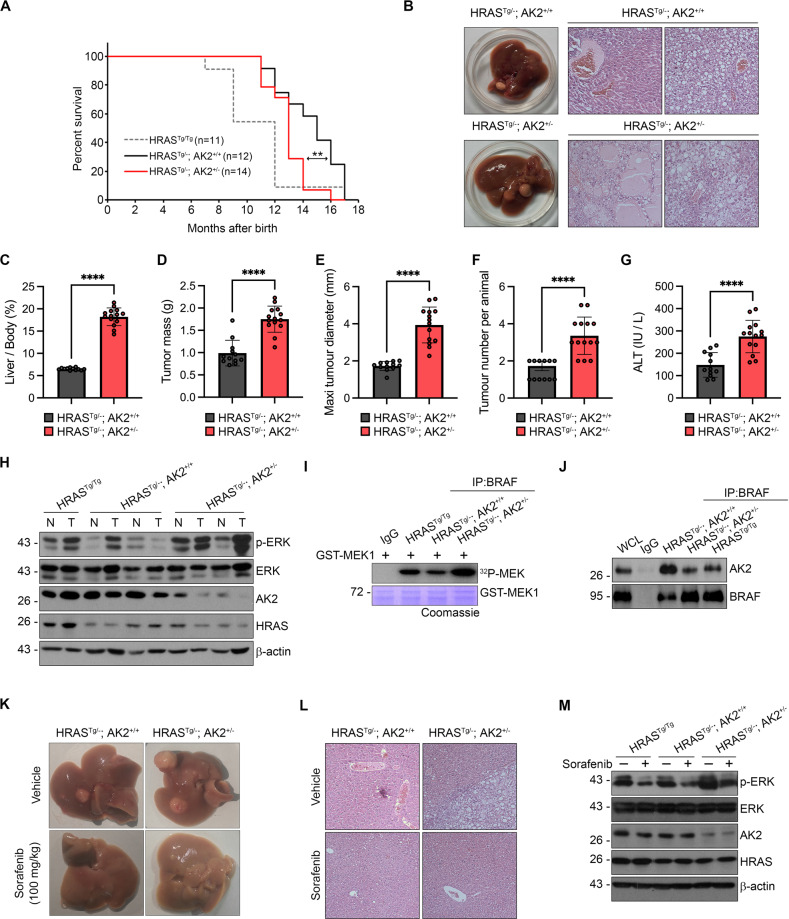
AK2 ablation exacerbates tumor formation in *HRAS*^G12V^mouse model of HCC. **A** Reduced survival of *HRAS*^*G12V+/−*^ mice by *AK2*^+/−^ deficiency. Survival curves of *HRAS*^*G12V+/−*^:*AK2*^*+/+*^ (*n* = 12, HRAS^Tg/−^; AK2^+/+^), *HRAS*^*G12V+/−*^:*AK2*^*+/−*^ (*n* = 14, HRAS^Tg/−^; AK2^+/−^), and *HRAS*^*G12V+/+*^ (*n* = 11, HRAS^Tg/Tg^) mice with median survival of 450, 360, and 300 days, respectively. **p = 0.001172 by log-rank test. **B** Pathology of liver tumor by *AK2*^*+/−*^ deficiency. Gross histology (left) and representative H&E staining of paraffin-embedded liver sections (right) obtained from *HRAS*^*G12V+/−*^:*AK2*^*+/+*^ (HRAS^Tg/−^; AK2^+/+^) and *HRAS*^*G12V+/−*^:*AK2*^*+/−*^ (HRAS^Tg/−^; AK2^+/−^*)* mice at 8 months of age. **C**–**G** High incidence of liver tumor by *AK2*^*+/−*^ deficiency. After tumors had grown to the approved size, the liver/body weight ratios (**C**), liver tumor volumes (**D**), maximal diameters (**E**), average tumor burden (**F**), and circulating blood levels of ALT (**G**) were quantified. Values are mean ± S.E.M. (*n* = 12, 14 for each *HRAS*^*G12V+/−*^:*AK2*^*+/+*^ and *HRAS*^*G12V+/−*^:*AK2*^*+/−*^; *p < 0.05, **p < 0.01, ***p < 0.001, ***p < 0.0001); paired Student’s t-test. **H**–**J** Combinations of *HRAS*^*G12V*^ and *AK2*^*+/−*^ alleles amplify the ERK activity. Liver tumor extracts of *HRAS*^G12V+/−^:*AK2*^+/+^ (HRAS^Tg/−^; AK2^+/+^), *HRAS*^G12V+/−^:*AK2*^+/−^ (HRAS^Tg/−^; AK2^+/−^), and *HRAS*^G12V+/+^ (HRAS^Tg/Tg^) mice in (**B**) were analyzed with western blotting (**H**) and immunoprecipitation (IP) assay (**J**) followed by kinase reaction with γ-^32^p-MEK1 (**I**). Total β-actin was used as a loading control. **K**−**M** Sorafenib retards tumor growth in 7-month-old *HRAS*^*G12V+/−*^:*AK2*^*+/−*^ mice. *HRAS*^G12V+/−^:*AK2*^+/+^ (HRAS^Tg/−^; AK2^+/+^) and *HRAS*^G12V+/−^:*AK2*^*+/−*^ (HRAS^Tg/−^; AK2^+/−^) mice were daily treated with 100 mg kg^−1^ body weight Sorafenib for 3 weeks. Gross appearances of livers lesions (**K**) and representative H&E staining of paraffin-embedded liver sections (**L**) were examined in four groups of the mice. Liver tumor extracts were subjected to western blotting (**M**). Total β-actin was used as a loading control.

To corroborate that the low levels of AK2 expression in liver tumors affect tumor development in vivo, we examined the effects of AK2 ablation on tumorigenesis in an established mouse model of liver cancer. Because a homozygous *AK2* knockout (*AK2*^−/−^) mice that we generated was embryonically lethal, we utilized heterozygous *AK2* knockout (*AK2*^+/−^) mice for analyses. We could not see any obvious phenotypic difference of the livers between WT and AK2^+/−^ mice up to 2 years. We crossed *AK2*^+/−^ mice with the *HRAS*^G12V^ transgenic (Tg) mouse model of HCC [37, 38], corresponding to the most frequently mutated form of *HRAS* found in human cancers [42]. HRAS^Tg/−^; AK2^+/−^ mice survived for a shorter period than did HRAS^Tg/−^; AK2^+/+^ mice. Median survival was 450 days for HRAS^Tg/−^; AK2^+/+^ mice and 360 days for HRAS^Tg/−^; AK2^+/−^ mice (Fig. 5A).

Next, 7–8 months-old mice were euthanized and analyzed for potential tumor formation in gross examination of liver tissues. Visible hepatic tumor foci developed in all groups but more tumors were observed in 32-week-old HRAS^Tg/−^; AK2^+/−^ mice in comparison with HRAS^Tg/−^; AK2^+/+^ mice (Fig. 5B, left). Hematoxylin and eosin staining (H&E) of the liver sections uncovered the presence of large areas of parenchymal necrosis and well-pronounced fat droplets in HRAS^Tg/−^; AK2^+/−^ mice but much less in HRAS^Tg/−^; AK2^+/+^ mice (Fig. 5B, right). *AK2* deletion significantly increased the relative liver weight and volumes, sizes, and numbers of liver tumors almost two-fold as compared to HRAS^Tg/−^; AK2^+/+^ controls (Fig. 5C–F). However, the weights of the body, kidneys, and heart, but not spleen, were indistinguishable from the controls (Fig. S5A–D). Consistently, expression of *HRAS*^G12V^ elicited greater liver damage in *AK2*^+/−^ mice than in WT controls, as indicated by high serum levels of alanine aminotransferase (ALT) and aspartate aminotransferase (AST) in *AK2*^+/−^ mice (Fig. 5G and Fig. S5E). In agreement with other report [43], HCC formation in HRAS-Tg mice was greater in males than in females (Fig. S6A–J). However, *AK2*^+/−^ in both HRAS-Tg mouse genders yielded a marked increase in the incidence of HCC. Collectively, the results suggest that the heterozygous loss of *AK2* sustains and enhances HRAS-induced tumorigenesis.

In addition, p-ERK levels were higher in most of liver tumors (T) than in nontumorous surrounding tissues (N) and this upregulation of p-ERK was larger in *AK2*^+/−^mouse tumors than in *AK2*^*+/+*^ tumors (Fig. 5H and Fig. S5F, G). BRAF activity was also reduced and AK2-BRAF interaction was enhanced in HRAS^Tg/−^; AK2^+/+^ mice compared to HRAS^Tg/−^; AK2^+/−^ mice (Fig. 5I, J).

To determine the contribution of the reinforced BRAF/MAPK to the formation of HCC in AK2-deficient mice, we examined antitumor effects of multi-kinase inhibitor, Sorafenib, which had been shown to also inhibit BRAF. Sorafenib was daily administered by intraperitoneal (i.p.) injection for 3 weeks and tumor formation was analyzed. Interestingly, Sorafenib administration led to near-complete tumor regression in HRAS^Tg/−^; AK2^+/−^ mice as effectively as in HRAS^Tg/−^; AK2^+/+^ mice (Fig. 5K). Sorafenib significantly attenuated the tumorigenic effect of AK2 ablation, including the invasive characteristics of HCC (Fig. 5L) and the activation of ERK (Fig. 5M). Thus, inhibition of BRAF/MAPK is important for the HRAS^G12V^-driven formation of HCC in AK2-deficient mice.

### AK2 interacts with BRAF in a RAS-dependent manner

**Fig. 6:**
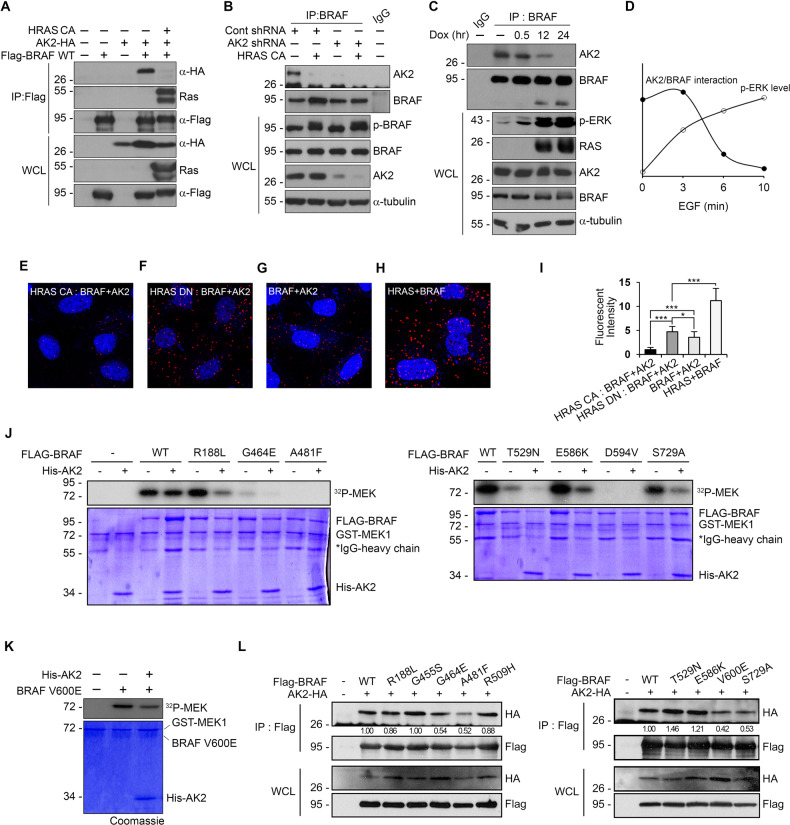
RAS activation impairs the AK2-BRAF interaction and AK2 restrains inhibitor-insensitive BRAF mutants. **A**–**C** RAS activation reduces the association of AK2 and BRAF. HEK293T cells were cotransfected with Flag-BRAF, AK2-HA, and either control vector (-) or constitutively active HRAS (HRAS CA) for 24 h and then subjected to immunoprecipitation (IP) analysis (**A**). Hep3B and Hep3B-shAK2 cells were transfected with activated HRAS (HRAS CA) and then subjected to immunoprecipitation (IP) analysis (**B**). NIH3T3 cells stably expressing HRAS^V12^ were treated with 0.5 μg ml^−1^ doxycycline for the indicated times and subjected to immunoprecipitation (IP) analysis (**C**). Total **ɑ**-tubulin was used as a loading control. **D** A graph showing the relative association between AK2 and BRAF in HeLa cells upon EGF (50 ng ml^−1^) treatment over the indicated period of time. **E**–**I** In situ proximity ligation assay showing the regulated interaction between AK2 and BRAF according to HRAS activity. HeLa cells were left untreated (**G**, **H**) or transfected with either HRAS-CA (**E**) or HRAS-DN (**F**), and then subjected to PLA and DAPI staining for visualization of nuclei (blue). Red dots indicate the protein–protein interaction between RAS and BRAF (**H**) and BRAF and AK2 (**E**–**G**). The intensities of red dots in the assays were quantified (**I**) (*n* = 7; different fields of microscopy; values are presented as mean ± SD (*n* = 3; *p < 0.05, **p < 0.01, ***p < 0.001). **J** AK2 reduces the kinase activity of BRAF mutants found in cancer patients. HEK293T cells were transfected with Flag-BRAF (WT) or cancer patient-found BRAF mutants. Flag-BRAF proteins were then immunopurified and assayed for the kinase activity using γ-^32^p-MEK1 in the absence or presence of His-AK2 protein. **K** In vitro modulation of BRAF^V600E^ activities by AK2. Purified AK2 protein (1 μM) was incubated with BRAF^V600E^ protein (10 nM). MEK phosphorylation was visualized by autoradiography. Proteins utilized in the assay were confirmed by western blotting and Coomassie staining. **L** Interaction of AK2-BRAF mutants. HEK293T cells were transfected with AK2-HA and Flag-tagged BRAF mutants for 24 h and subjected to immunoprecipitation (IP) assays using Flag antibody. The immunoprecipitates and whole cell lysates (WCL) were analyzed by western blotting. Ratio of [anti-HA; FLAG-IP] to [anti-HA; WCL] on each BRAF mutant are represented in the blots.

We hypothesized that RAS activation affected AK2–BRAF interaction as the interaction was reduced in HRAS^Tg/Tg^ mice. Expression of CFP-HRAS^G12V^ (HRAS CA) indeed caused dissociation of AK2 from BRAF (Fig. 6A, B). Similarly, we confirmed the effect in NIH3T3 cells stably expressing HRAS^G12V^ via a Tet-on system (Fig. 6C). Moreover, EGF, which is known to activate RAS, caused the dissociation of AK2 from BRAF and triggered concomitant activation of ERK at the same time points (Fig. 6D). Using in situ proximity ligation assays, we visualized the intracellular interaction between AK2 and BRAF dependent on RAS activity. RAS–BRAF complex showed punctate signals and AK2-BRAF complex were also detected (Fig. 6G, H). As expected, the AK2–BRAF interaction was strengthened by the expression of the dominant negative HRAS mutant (HRAS DN) (Fig. 6F, I) but weakened by the activated HRAS (HRAS CA) (Fig. 6E, I). Together, these results suggest that RAS activation causes a release of BRAF from AK2 and concomitant activation of BRAF and downstream ERK.

### Activity of BRAF mutants found in cancer patients is inhibited by the AK2

Various BRAF mutations including BRAF^V600E^ and BRAF^S729A^ have been identified in cancer patients, such as lung adenocarcinoma and melanomas [44, 45]. Therefore, we advanced to examine whether AK2 inhibited the kinase activity of these BRAF mutants. In in vitro kinase assays of the immunopurified FLAG-BRAF mutant proteins, we found that kinase activities of these BRAF mutants were suppressed by addition of the AK2 protein (Fig. 6J). To exclude the background level of ^32^P incorporation into BRAF for the inhibition of enzyme activity due to BRAF autophosphorylation, BRAF kinase-dead (D594V) and an ATP-binding-deficient (A481F) mutants were also included and evaluated as controls in these assays; this inhibition by AK2 is distinct from BRAF autoinhibition. Also, the addition of AK2 protein to the in vitro reaction a little suppressed the enzymatic activity of the BRAF V600E mutant (Fig. 6K). Additionally, immunoprecipitation assays revealed that most BRAF mutants showed similar binding affinities to AK2, but BRAF^G464E^, BRAF^A481F^, BRAF^V600E^, and BRAF^S729A^ mutants showed reduced binding to AK2 compared to BRAF^WT^ (Fig. 6L). Totally, enhancing the AK2-BRAF interaction in tumor cells, such as inducing energy deprivation, might provide one strategy to combat BRAF dysfunction.

## Discussion

Generally, in cancers, metabolic dysregulation is accompanied by alteration of the AMP/ATP ratio rather than ATP or ADP concentration [46, 47]. These data emphasize the crucial function of AK2 in BRAF regulation and tumorigenesis under low energy state. More, given that intracellular AMP levels may be a pivotal determinant of drug resistance [48, 49] as well as tumorigenesis, it should be interesting to examine AK2 levels in tumor cells manifesting drug resistance. Consequently, the question how low energy/AMP can amplify AK2 function in BRAF regulation is an important one. As evidenced by the enhanced affinity of the AK2–AMP complex for BRAF, it is conceivable that the binding of AMP to AK2 induces conformational changes of AK2 that increase its affinity for BRAF. AK2 has been reported to switch to the fully closed conformation by AMP [48, 50–52]. Similarly, our results suggest that the NMP domain of AK2 is crucial for the binding to BRAF and regulation of BRAF activity. Furthermore, our in vitro assays showed that the AK2 reduced autophosphorylation of BRAF WT (Fig. S7A). Holderfield et al. reported RAF inhibitors promote tumor growth by relieving RAF autoinhibition [53]. It was reported that BRAF G464E and BRAF G464V disrupt P-loop autoinhibition and BRAF V600E is insensitive to P-loop autoinhibition [53]. By contrast, the kinase activities of BRAF G464E and V600E were a little regulated by AK2, suggesting that the AK2 may restrain the BRAF kinase activity by inhibiting BRAF autophosphorylation, which needs to be further determined.

**Fig. 7:**
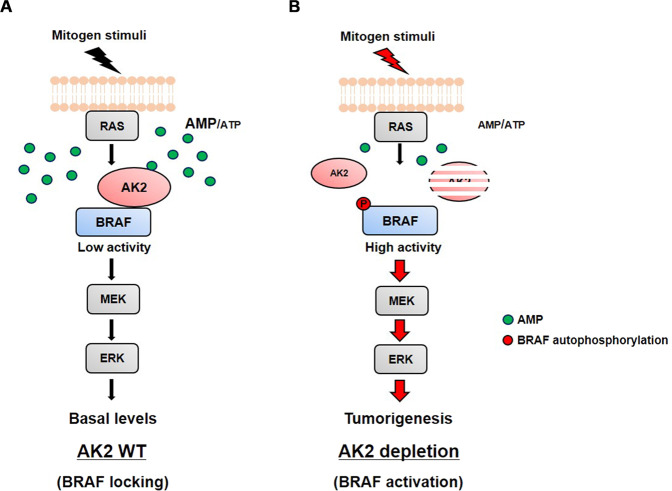
Proposed model of AK2 function as a BRAF locker in the RAS-BRAF signaling. **A** Under resting condition with high AMP level, AK2 senses AMP and forms a protein complex with BRAF to inhibit the BRAF activity. **B** Upon mitogen stimulation and RAS activation, AK2 is released from BRAF, leading to activation of BRAF.

An intriguing possibility that AK2 regulates the BRAF-driven tumorigenesis was addressed by means of *HRAS*-Tg and *AK2*^+/−^ mice. We also found that the AK2 level was largely reduced in 86.8% of human HCC tissue samples. In accordance to this, it was reported that AK2 is downregulated in a mouse model of HCC triggered by a liver-specific double KO of *PTEN* and *TSC1* [54]. In TCGA, we identified 67 AK2 mutations found in the uterus (25, 5.85%), skin (10, 1.92%), colon (2, 1.46%), thyroid (6, 0.64%), and liver (13, 0.6%). The types of mutations encompass missense and nonsense mutations as well as deletions, resulting in a massive loss of the AK2 protein in patients with cancer. Thus, the loss of AK2 is possibly involved in the pathogenesis of not only HCC, but other types of cancer (Fig. 7).

Almost 200 BRAF mutants have been identified in various human cancers [55, 56]. Activating BRAF mutations, in particular V600E, drive the formation of many types of cancer, whereas inactivating mutations or WT BRAF cooperate with RAS via paradoxical ERK activation and cause resistance to the BRAF-selective inhibitors. Notably, we found that AK2 inhibited the kinase activities of various BRAF mutants. Even though AK2 showed tumor-suppressive roles and low AK2 expression correlated with poor prognosis of HCC patients, further studies focusing on the role of AK2 expression and AMP in HCC are required to offer possible therapeutic modalities in not only BRAF-driven cancers but cancers developing resistance to BRAF inhibitors.

## Supplementary information

Supplementary Information

Author checklist

## Data Availability

The Cancer Genome Atlas (TCGA) HCC microarray dataset and clinical information were obtained from ‘Firehose’ (gdac.broadinstitute.org). GSE14520 (based on the GPL3921 platform) HCC- and normal tissue- datasets and clinical information were downloaded from ‘NCBI Gene Expression Omnibus’ (www.ncbi.nlm.nih.gov/geo).
