# Supplementary material for: AK2 is an AMP-sensing negative regulator of BRAF in tumorigenesis
Source: Cell Death Dis. 2022 May 18;13(5):469. doi: 10.1038/s41419-022-04921-7 (PMC9117275; doi:10.1038/s41419-022-04921-7)
Supplement: Supplementary file 1 — Supplementary Information [file 41419_2022_4921_MOESM1_ESM.docx]

**Supplementary Information**

**Supplementary materials and methods**

**Cell culture and transfection**

Cells were authenticated by STR profiling at ATCC, JCRB and KCLB and showed negative results on mycoplasma contamination tests. Cells were used in the assays within 10 passages after collected from ATCC, JCRB and KCLB. HEK293T, Chang liver, Hep3B, HepG2, SK-Hep1, SK-Mel-2, and DLD-1 cells were obtained from ATCC; HLE and Huh7 cells were obtained from JCRB; SNU-182, SNU-354, SNU-368, SNU-387, SNU-398, SNU-423, SNU-449, NCTC-1469 and SNU-475 cells were obtained from Korean Cell Line Bank (KCLB). DLD-1 and NIH3T3/HRAS^G12V^ inducible cells were gifts from Dr. K.Y. Choi (Yonsei Univ. Seoul, Korea). All the cells were cultured in Dulbecco’s Modified Eagles Medium (DMEM) or Roswell Park Memorial Institute Medium (RPMI) (HyClone, South Logan, UT) supplemented with 10% fetal bovine serum (FBS; HyClone) and 1% penicillin/streptomycin. WT and *AK2* knockout MEFs were cultured with DMEM with 10% FBS. Transfection was carried out with LipofectAMINE reagent (Invitrogen, Carlsbad, CA) following the manufacturer’s instructions. For generation of stable cell lines, Hep3B and HLE cells were transfected with vector using LipofectAMINE reagent (Invitrogen) for 24 h and then grown in selection medium containing 1 mg/ml G418 (Invitrogen) for 2 weeks.

**Plasmid constructs**

Construction of AK2 (pAK2-HA, pFLAG-AK2, pEGFP-AK2, pAK2 shRNA, and deletion mutants) was previously described (Kim et al., 2014; Lee et al., 2007). The cDNAs encoding BRAF were obtained by PCR and cloned into pcDNA3.1-HA (Addgene Plasmid #128034) and p3xFLAG-CMV-10 vectors (Sigma Aldrich Cat# E7658). The pECFP-HRAS constitutive active (CA) and dominant negative (DN) were provided by Dr. W.D. Heo (Daejeon, Korea). Point mutations of BRAF (R188L, G464E, A481F, T529N, E586K, D594V, and S729A) and AK2 (K28E, T46S, R51K, T46S/R51K, Q107N, R150A, R175K, and R186K) were generated by site-directed mutagenesis and verified by DNA sequencing analysis.

**Antibodies**

Polyclonal rabbit anti-AK2 (Santa Cruz Biotechnology Cat# sc-28786, RRID:AB_2225292) antibody was described previously (Kim et al., 2014). Anti-AK3 (Santa Cruz Biotechnology Cat# sc-398571), anti-phospho-BRAF (Cell Signaling Technology Cat# 2696, RRID:AB_390721), anti-BRAF (Millipore Cat# 04-328, RRID:AB_11204532), anti-CRAF (Santa Cruz Biotechnology Cat# sc-133, RRID:AB_632305), anti-phospho-ERK (Cell Signaling Technology Cat# 9101, RRID:AB_331646), anti-ERK (Cell Signaling Technology Cat# 9102, RRID:AB_330744), anti-phospho-MEK1/2 (Ser217/221) (Cell Signaling Technology Cat# 9121, RRID:AB_331648), anti-phospho-Elk-1 (Santa Cruz Biotechnology Cat# sc-8406, RRID:AB_627509), anti-Elk-1 (Santa Cruz Biotechnology Cat# sc-355, RRID:AB_631429), anti-GFP (Santa Cruz Biotechnology Cat# sc-8334, RRID:AB_641123), anti-GAPDH (Santa Cruz Biotechnology Cat# sc-59541, RRID:AB_783594), anti-α-tubulin (Santa Cruz Biotechnology Cat# sc-23948, RRID:AB_628410), and anti-Flag M2 (Sigma-Aldrich Cat# F1804, RRID:AB_262044) antibodies were used in western blot analysis.

**GST pull-down assay**

Purified GST-fusion proteins coupled to Glutathione-Sepharose 4B (GE Healthcare Cat# 17-0756-01) were incubated for 3 h in binding buffer (19) at 4 °C with purified His-fusion proteins or [^35^S]-methionine-labeled proteins which were *in vitro* translated using a TNT-coupled transcription/translation system (Promega Cat# L1170). After pull-down assay with Glutathione-Sepharose beads, the precipitated proteins were extensively washed with binding buffer, separated by 10% SDS-PAGE, and detected by western blotting and autoradiography.

**Cell proliferation assay**

To measure cell density using DNA content, 1 × 10^4^ cells were seeded into 96-well plates, after which cells were removed every day and immediately frozen at -70 °C. Cells were then thawed and lysed, and DNA content was measured using a CyQUANT® Cell Proliferation Assay Kit (Thermo Fisher Scientific Cat# C7026) by measuring the fluorescent signal (excitation, 480 nm; emission, 520 nm).

***In situ* PLA assay**

The *in situ* PLA was performed on fixed cells with DuoLink PLA technology probes and reagents (Sigma Aldrich Cat# DUO92101), and following the manufacturers protocol. First, cells were permeabilized with 0.3% PBST for 15 min. After washed with PBS, cells were incubated with blocking solution for 1 h at 37 °C and then with the primary antibodies for overnight at 4 °C. Every experiment was performed with a pair of antibodies of different species. The cover slips were washed with buffer A, followed by incubation with the PLA probes (secondary antibodies against two different species bound to two oligonucleotides: anti-mouse MINUS and anti-rabbit PLUS) in antibody diluent for 60 min at 37 °C. After two washes of 5 min with buffer A, the ligation step was performed for 30 min at 37 °C. Cells were washed with buffer A twice for 2 min before incubation for 2 h with amplification stock solution at 37 °C. After two washes of 10 min with buffer B, cells were incubated with Alexa Fluor 488 and 594 (Jackson). Finally, cover slips were washed with PBS and mounted with Duolink *in situ* mounting medium containing DAPI.

**Animal studies**

At the end of the experiment, mice were sacrificed and livers were collected, and tumor numbers and sizes on the surface were then evaluated. The histological analysis was performed in formalin-fixed, hematoxylin- and eosin-stained liver sections. For Sorafenib administration, 6-month-old mice were daily treated with 100 mg/kg body weight Sorafenib in a total volume of 200 μl for 3 weeks. Sorafenib was dissolved in 4x cremophor EL/95 % ethanol solution (50:50). After tumors had grown to the distinguishable size but before their largest tumor diameter had exceeded 20 mm, tumors were dissected and their volumes, numbers, and diameters were measured. Survival of mice are statistically analyzed by log-rank test (n = 11, 12, 14). Statistical analysis on liver and tumor phenotypes of mice was assessed by paired Student’s t-test (n = 12, 14). All experiments were done in an age and sex-controlled fashion unless otherwise noted in the figure legends. No data were excluded from mice analysis in the study. Detailed information for animal models and habituation condition is described below; species - Mus Musculus; Strain - C57BL/6J; gender - male and female; age - 1 to 18 months old. All mice were housed and bred in specific pathogen-free facility maintaining temperature at 23 °C.

**Human HCC analysis**

Whole human liver tissues or 3 mm-thick sections of formaldehyde-fixed and paraffin-embedded samples of a non-selected cohort of surgically resected HCCs were obtained from the Biobank of Chonbuk National University Hospital, a member of the National Biobank of Korea, which is supported by the Ministry of Health, Welfare and Family Affairs. All samples derived from the National Biobank of Korea were obtained with informed consent under institutional review board-approved protocols. National Biobank of Korea obtained "informed written consent" from the subjects. No statistical methods were used to predetermine sample size, but the sample size analyzed in this study is similar to the previous publications (e.g. [29]). Patients are 43-86 years of age (median age is 63) and both male and female patients are included (male – 41; female – 12). No relevant diagnosis was performed. No data were excluded from human HCC analysis in the study.

The Cancer Genome Atlas (TCGA) HCC microarray dataset and clinical information were obtained from ‘Firehose’ (gdac.broadinstitute.org). GSE14520 (based on the GPL3921 platform) HCC- and normal tissue- datasets and clinical information were downloaded from ‘NCBI Gene Expression Omnibus’ (www.ncbi.nlm.nih.gov/geo).

**Statistical analysis**

Data presented are mean ± SEM (n = 3), with all replicates in intact cell experiments from separate dishes of cells. All animal experiments were performed comparing littermates, the evaluators were aware of animal identity throughout the experiments and outcome assessment. The log-rank test was used to evaluate the significance of the difference in survival. Unless stated otherwise, statistical analysis (GraphPad Prism 5 for Mac OSX) was by ANOVA, using Bonferroni’s multiple comparison test of selected data sets (**p* < 0.05, ***p* < 0.01, ****p* < 0.001; ns, not significant).

**Supplementary Table 1. Candidate BRAF- or AK2-binding proteins identified by LC-MS/MS.** Immunoprecipitated proteins by anti-Flag antibody-conjugated agarose bead were trypsinized and analyzed using LC-MS/MS. The number of identified peptides for the proteins are shown in table.

|  | **Reference** | **Number of identified peptides** |
| --- | --- | --- |
| **BRAF-binding proteins** | **BRAF** | 49 |
|  | 14-3-3 *beta/alpha* | 27 |
|  | TY3H | 24 |
|  | 14-3-3 *zeta/delta* | 22 |
|  | RAF-1 | 18 |
|  | 14-3-3 *eta* | 15 |
|  | GRP78 | 15 |
|  | **AK2** | 13 |
|  | HSP71 | 10 |
|  | 14-3-3 *gamma* | 9 |
|  | HSP70 | 8 |
|  | TPM4 | 8 |
|  | HSP90 | 7 |
| **AK2-binding proteins** | **AK2** | 82 |
|  | VIMENTIN | 77 |
|  | ACTBL2 | 53 |
|  | POTEE | 43 |
|  | **BRAF** | 23 |
|  | MYH9 | 17 |
|  | DBN1 | 14 |
|  | HSP71 | 13 |
|  | HSP70 | 12 |
|  | MYO1C | 12 |
|  | JUP | 8 |
|  | TUBULIN *alpha* | 8 |
|  | HSP60 | 7 |

**Supplementary figures**

**
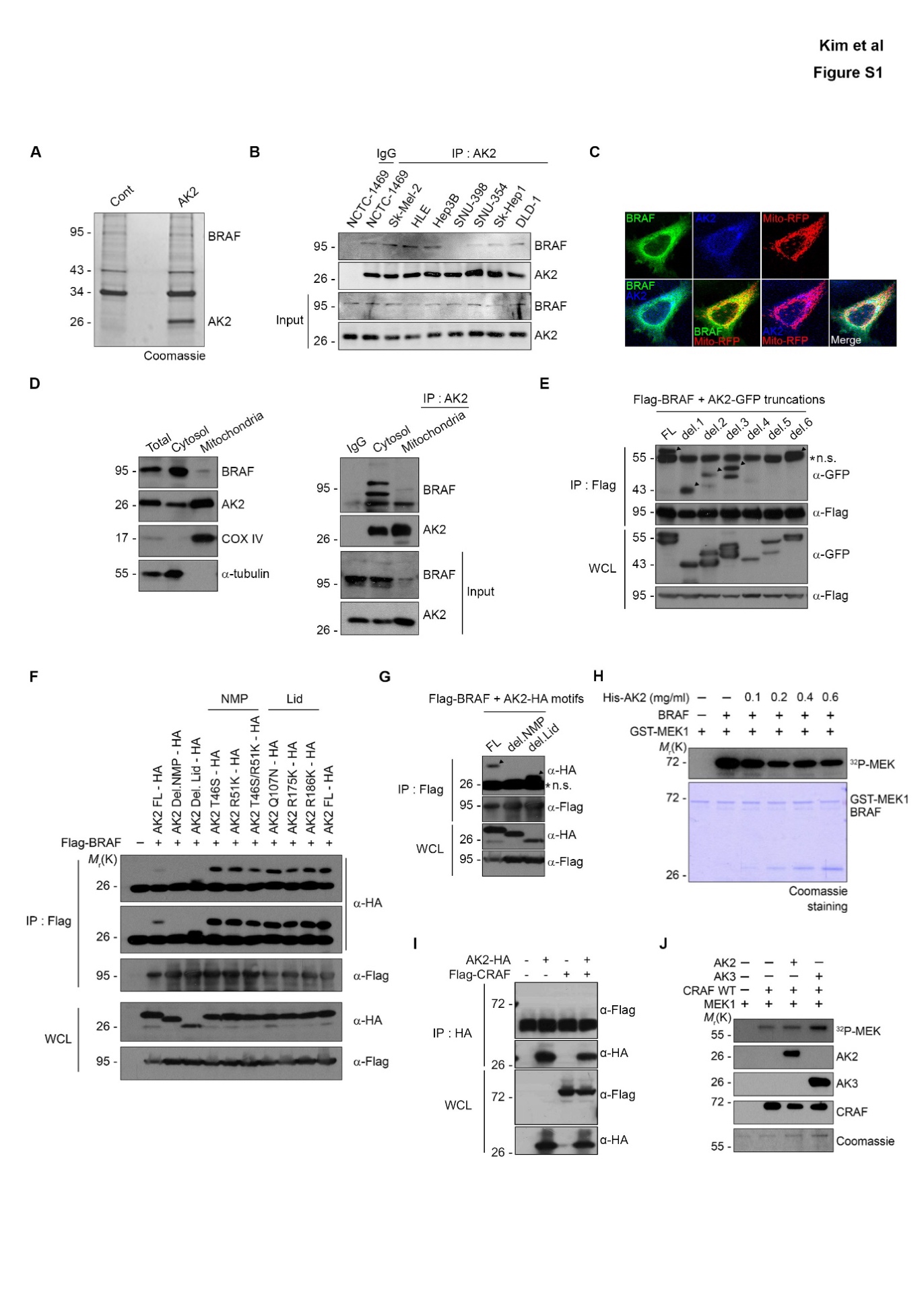
**

**Figure S1. Isolation of BRAF as a binding protein to AK2.**

**(A)** Flag-AK2 complexes isolated from HEK293T cells were subjected to mass spectrometry analysis.

**(B)** AK2 interacts with BRAF in cancer cell lines harboring BRAF WT. BRAF WT tumor cell lines and NCTC-1469 normal hepatocyte cell line were analyzed by immunoprecipitation (IP) assay using anti-AK2 antibody or IgG. The immunoprecipitates and input containing the same amount of AK2 protein were proved by western blotting.

**(C)** The subcellular localizations of endogenous BRAF (green) and AK2 (blue) in HeLa cells transiently expressing Mito-RFP (red) was examined by immunocytochemical analysis. Each signal (upper) and the overlaps of two or three (Merge) signals are shown (lower).

**(D)** AK2 binds to BRAF in the cytosol. Hep3B cells were fractionated into the cytosol and mitochondria-enriched fractions by centrifugation and the fraction was analyzed by western blotting (left) or by immunoprecipitation (IP) assay (right). COXIV and α-tubulin were served as markers for the cytosol and mitochondria, respectively.

**(E-F)** The N-terminal region of AK2 is required for its binding to BRAF. HEK293T cells were co-transfected with Flag-BRAF and GFP-tagged AK2 deletion mutants (E) or HA-tagged AK2 mutants (F) for 24 h, after which cell extracts were subjected to immunoprecipitation (IP) assay.

**(G)** The NMP domain of AK2 is indispensable for its interaction with BRAF. HEK293T cells were co-transfected with Flag-BRAF and HA-tagged AK2 del.NMP, AK2 del.Lid, or WT AK2 (FL), and subjected to immunoprecipitation (IP) assay.

**(H)** *In vitro* assay showing suppression of the BRAF kinase activities by AK2 in a dose-dependent manner. Purified AK2 protein (1 μM) was incubated with BRAF protein (10 nM). MEK phosphorylation was visualized by autoradiography. Proteins utilized in the assay was confirmed by western blotting and Coomassie blue staining

**(I)** AK2 does not bind to CRAF. HEK293T cells were co-transfected for 24 h with pcDNA3 (-), AK2-HA and Flag-CRAF as indicated and subjected to immunoprecipitation (IP) assays.

**(J)** AK2 does not inhibit CRAF kinase activity. GST-CRAF (10 nM) was incubated with either purified His-AK2 (1 μM) or His-AK3 (1 μM) protein. *In vitro* activity assay was performed as described in Figure 1E. The reaction products were separated by SDS-PAGE and exposed to X-ray film (upper) or proved by western blotting (lowers).

**
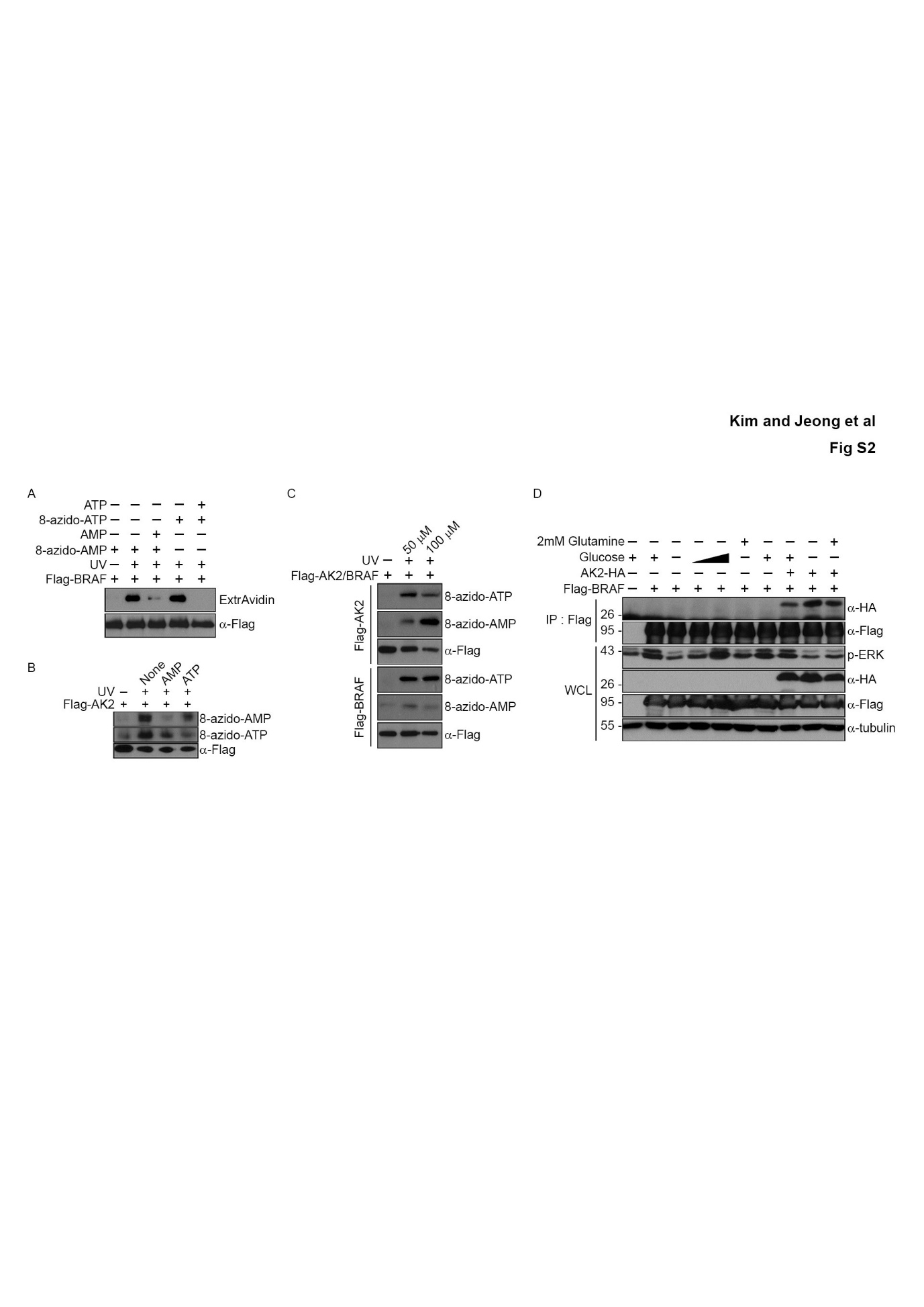
**

**Figure S2. Binding preference of BRAF and AK2 to ATP and AMP, respectively.**

**(A-C)** HEK293T cells transfected with Flag-BRAF (A), Flag-AK2 (B), or both Flag-BRAF and Flag-AK2 (C) were labeled with 100 μM 8-azido-ATP-biotin or 8-azido-AMP-biotin in the presence or absence of 1 mM non-photoreactive ATP or AMP. After UV irradiation, the reactions were separated by SDS-PAGE and 8-azido-ATP or 8-azido-AMP-labeled proteins were visualized by ExtrAvidin-HRP.

**(D)** Glucose deprivation enhances the AK2-BRAF interaction. HEK293T cells transfected with AK2-HA and Flag-BRAF were incubated with glucose-free medium or 2 mM glutamine medium for 6 h. Cell lysates were then immunoprecipitated (IP) with anti-Flag antibody, followed by western blotting. Total 𝛂-tubulin was used as a loading control.

**
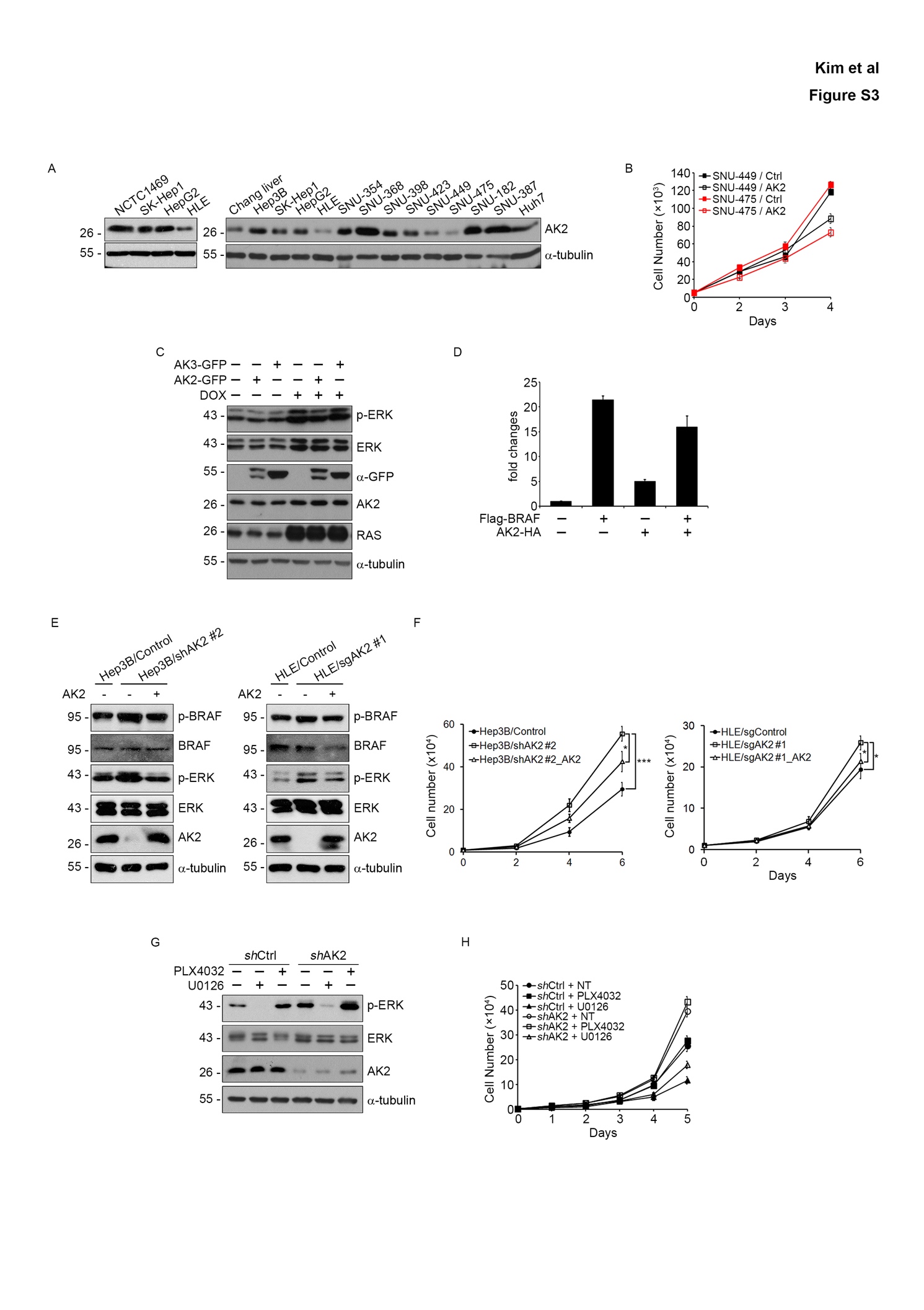
**

**Figure S3. AK2 exhibits anti-proliferative effect via EGF/RAS-induced BRAF signaling.**

**(A)** AK2 expression is down-regulated in liver cancer cell lines. The expression profile of AK2 was examined in a panel of HCC cell lines by western blot analysis and compared to NCTC1469 normal hepatocyte cell line. Total 𝛂-tubulin was used as a loading control.

**(B)** Reconstitution with AK2 decreases the rate of cell proliferation in hepatocellular carcinoma cell lines. SNU-449 and SNU-475 cells were transfected with either pcDNA or AK2-HA and cell proliferation was monitored. Values represent mean ± SD (n = 3).

**(C)** Ectopic expression of AK2, but not AK3, attenuates the RAS-induced ERK activation. NIH3T3 cells expressing the active RAS (HRAS^G12V^) in the presence of 100 ng ml^-1^ doxycycline were transfected with AK2-GFP or AK3-GFP, after which cell extracts were subjected to western blotting. Total 𝛂-tubulin was used as a loading control.

**(D)** AK2 interferes the BRAF-stimulated Elk-1 activity. HEK293T cells were co-transfected with BRAF, ELK-1/Gal-4, Gal4-E1b/luciferase, and control vector or AK2-HA. Then, Elk-1 activation was monitored by measuring luciferase activity. Values represent mean ± SD (n = 3).

**(E, F)** Reconstitution of AK2 in AK2-depleted Hep3B and HLE cells restrains the BRAF-ERK signaling and cell proliferation. AK2-depleted Hep3B/shAK2 #2 and HLE/sgAK2 #1 cell lines used in (Figure 3C−G) were transfected with pcDNA or AK2, and cell proliferation was monitored (F). The expression profiles of proteins were examined by western blot analysis (E). Total 𝛂-tubulin was used as a loading control. Values represent mean ± SD (n = 3). (**p* < 0.05, ***p* < 0.01, ****p* < 0.001).

**(G, H)** The ability of AK2 to regulate cell growth is dependent on MAPK signaling. Hep3B/Cont and Hep3B/AK2 shRNA cells (10^4^) per well in 6-well plates were cultured for 5 days with or without various MAPK kinase inhibitors (10 μM U0126 and PLX4032). The numbers of cells were then counted at the indicated days (H). The expression profiles of proteins were examined by western blot analysis (G). Total 𝛂-tubulin was used as a loading control.

**
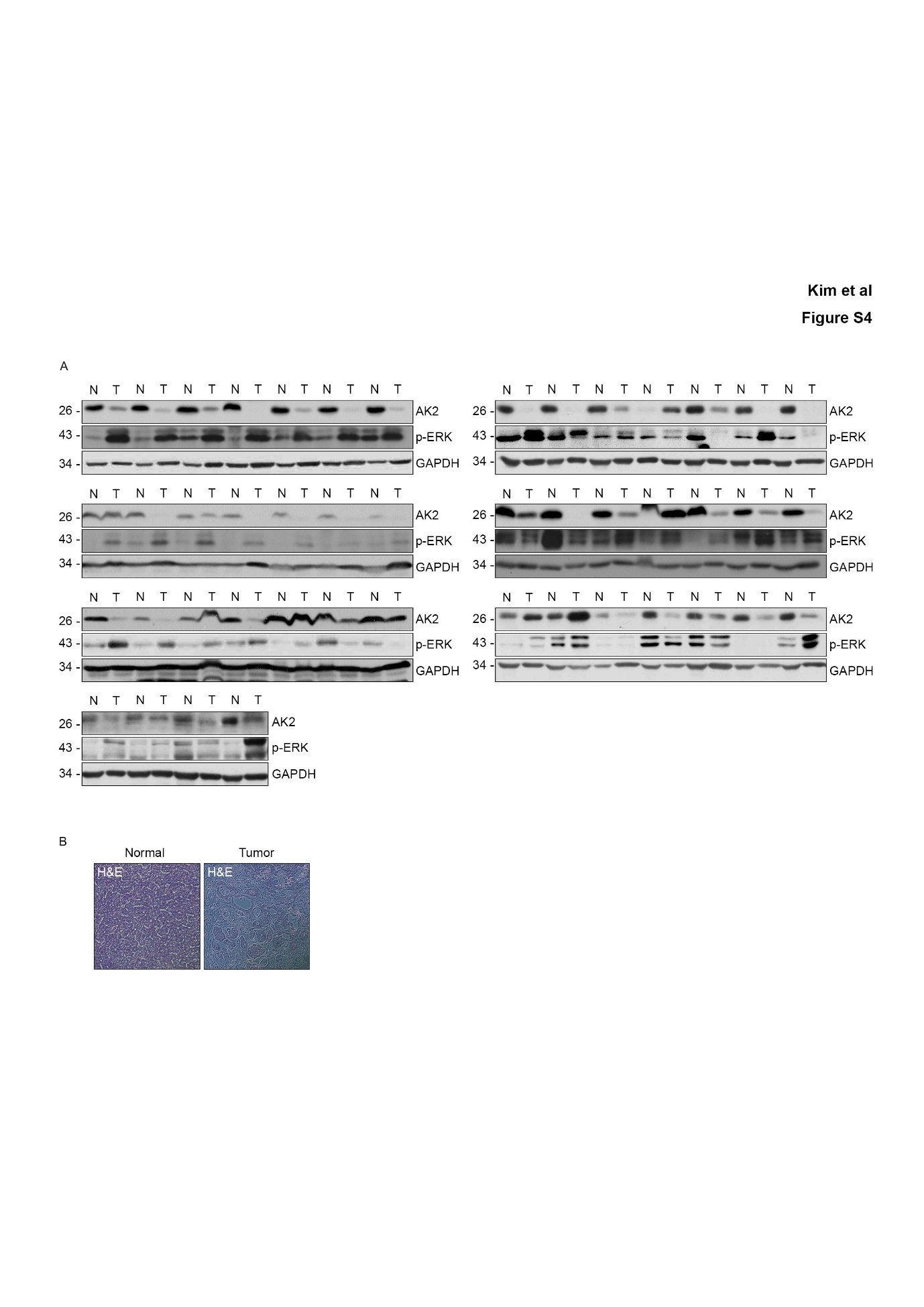
**

**Figure S4.** **AK2 is downregulated in most of human liver tumor tissues**

**(A)** AK2 expression is decreased in liver cancer specimens showing enhanced p-ERK. Total 53 normal liver (N) and cancer (T) samples were analyzed with western blotting. Total GAPDH was used as a loading control.

**(B)** Hematoxylin and eosin (H&E) staining on the paraffinized sections of tumor sample used in Figure 4G.

**
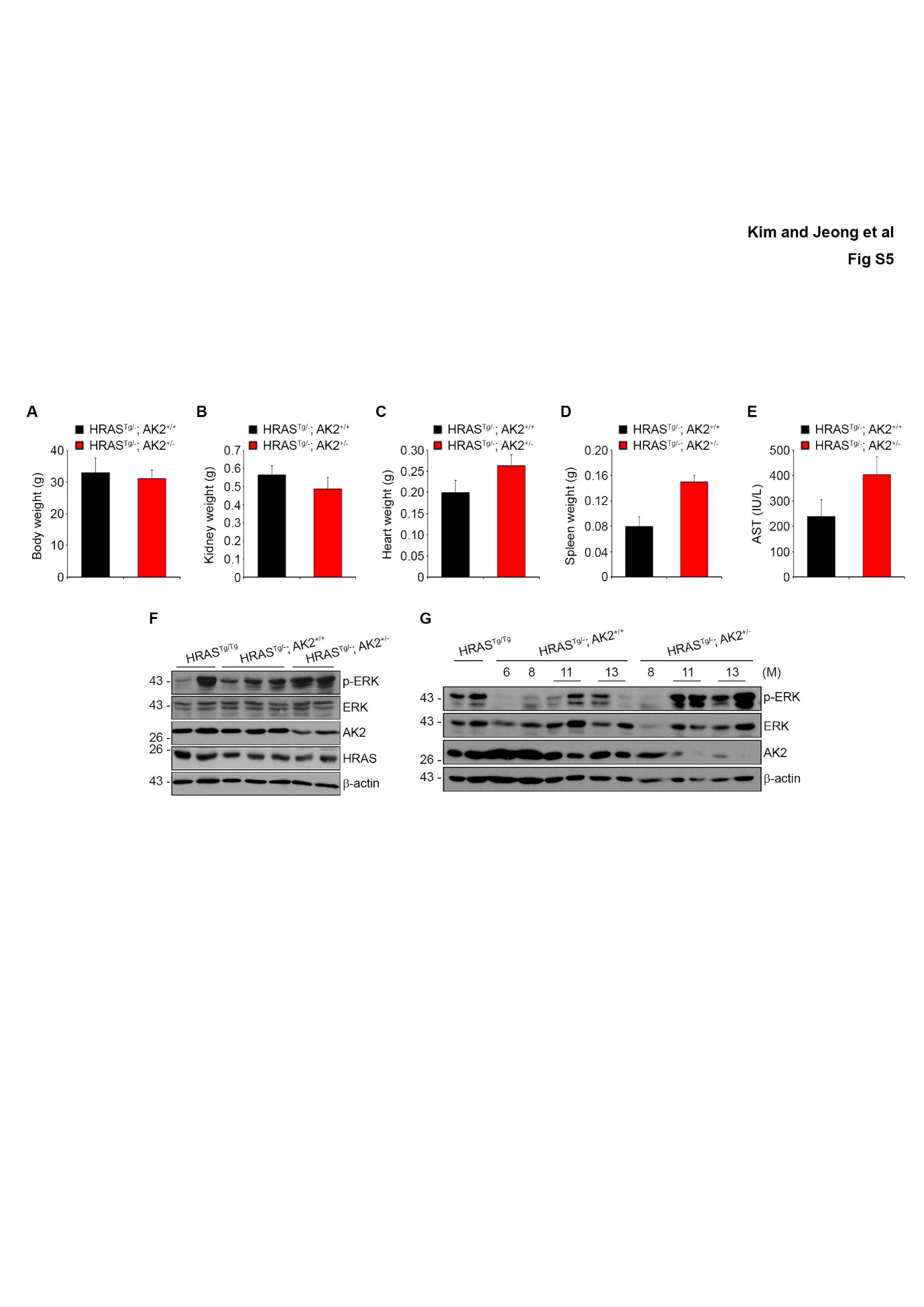
Figure S5. *AK2* deletion enhances tumor formation in the *HRAS^G12V^* mouse model of HCC.**

**(A-E)** After tumors had grown to the approved size, tumor tissues obtained from *HRAS*^G12V+/-^:*AK2*^+/+^ (HRAS^Tg/-^:AK2^+/+^) and *HRAS*^G12V+/-^:*AK2*^+/-^ (HRAS^Tg/-^:AK2^+/-^) mice at 12 months of age were examined for the weight of body (A), kidney (B), heart (C), spleen (D), and AST levels (E). Values are mean ± S.E.M. (n = 30).

**(F, G)** Enhanced level of p-ERK in the liver tumors of *AK2*-deficient mice. Liver tumor extracts were prepared from *HRAS*^G12V+/-^:*AK2*^+/+^ (HRAS^Tg/-^:AK2^+/+^), *HRAS*^G12V+/-^:*AK2*^+/-^ (HRAS^Tg/-^:AK2^+/-^), and *HRAS*^G12V+/+^ (HRAS^Tg/Tg^) mice at each month and analyzed with western blotting. Total β-actin was used as a loading control.

**
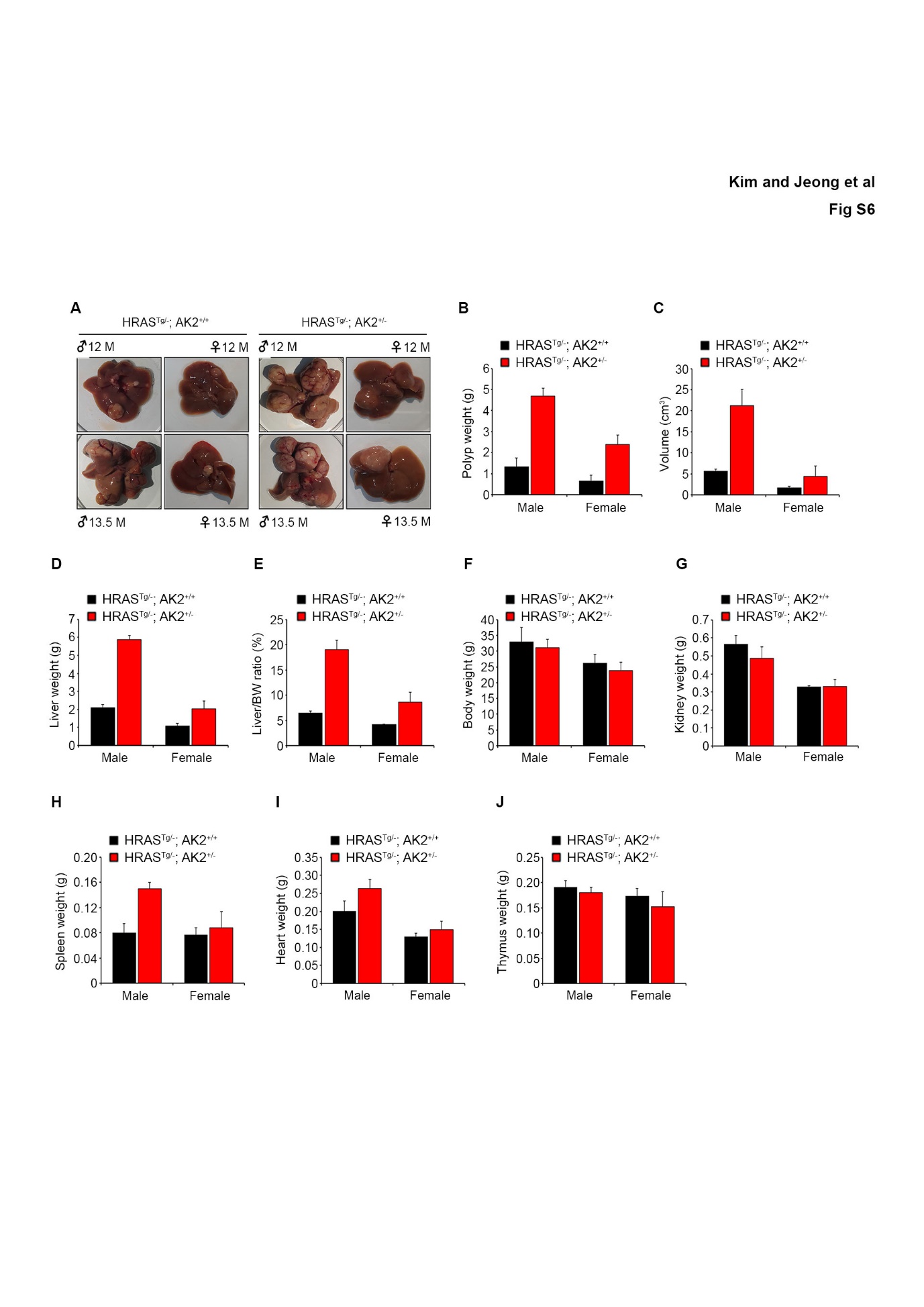
Figure S6. *AK2*-deficient mice expressing *HRAS^G12V^* show exacerbated tumor development in both female and male**

**(A)** *HRAS*^G12V+/-^:*AK2*^+/-^ mice display more liver tumor formation at 12 and 13.5 months in male and female mice. Gross histology of livers isolated from *HRAS*^G12V+/-^:*AK2*^+/+^ mice (male and female) and *HRAS*^G12V+/-^:*AK2*^+/-^ mice (male and female) were examined (n = 40).

**(B-J)** Pathologic parameters in the liver tumors of *HRAS*^G12V+/-^:*AK2*^+/-^ mice. After tumors had grown to the approved size, nodules grossly visible in the liver were quantified for polyp weight (B) and volume (C), liver weight (D), liver/BW ratio (E), and the weights of body (F), kidney (G), spleen (H), heart (I), and thymus (J).

**
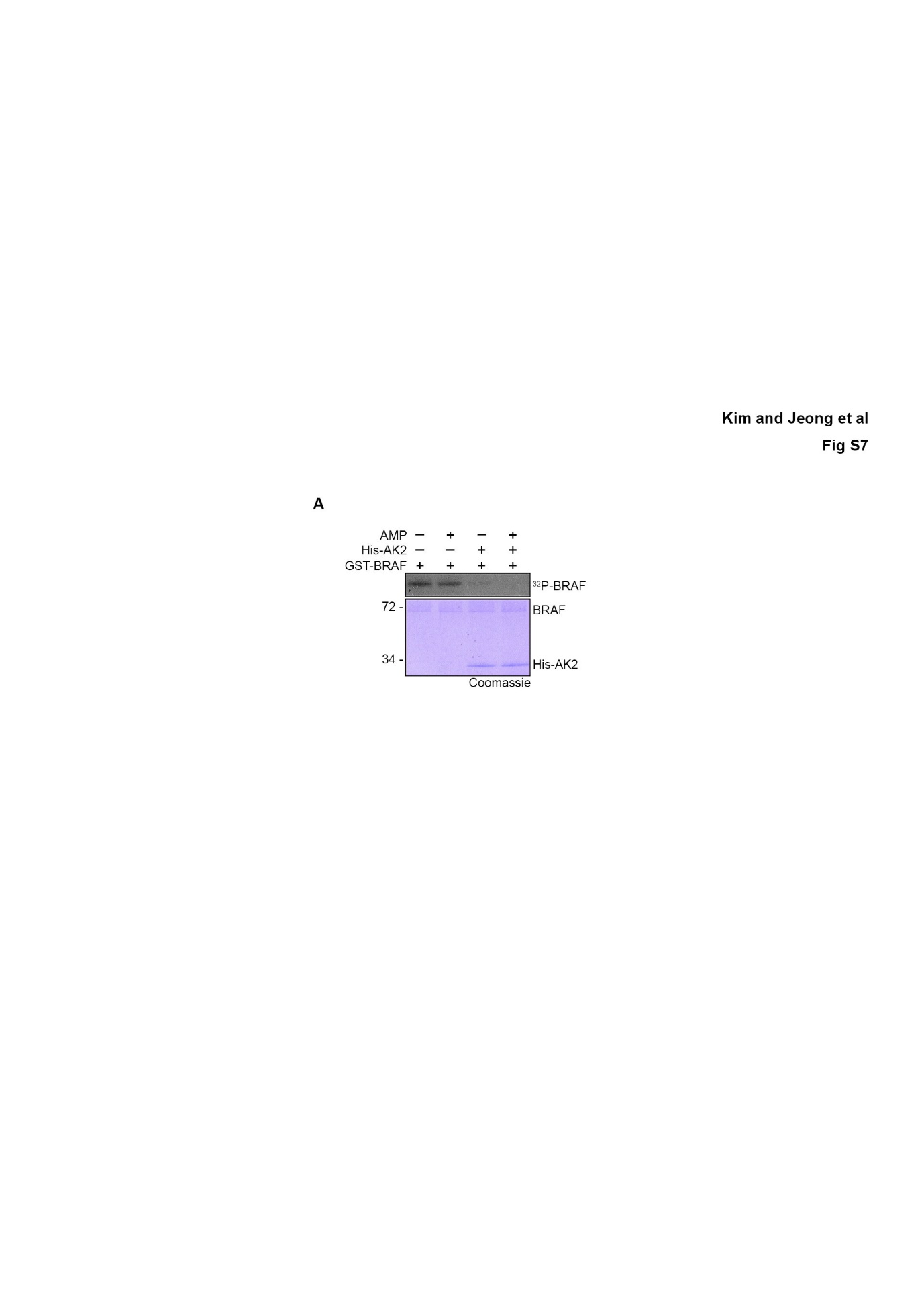
Figure S7. AK2 might disrupt BRAF phosphorylation.**

**(A)** Purified BRAF protein were incubated with or without AK2 protein. Then, the reaction products were separated by SDS-PAGE and visualized by autoradiography (^32^p-BRAF) or Coomassie blue staining (BRAF, His-AK2).
